# The lipid transfer protein OsLTPL159 is involved in cold tolerance at the early seedling stage in rice

**DOI:** 10.1111/pbi.13243

**Published:** 2019-09-11

**Authors:** Jie Zhao, Shanshan Wang, Jingjing Qin, Chuanqing Sun, Fengxia Liu

**Affiliations:** ^1^ State Key Laboratory of Plant Physiology and Biochemistry National Center for Evaluation of Agricultural Wild Plants (Rice) MOE Laboratory of Crop Heterosis and Utilization Beijing Key Laboratory of Crop Genetic Improvement Department of Plant Genetics and Breeding China Agricultural University Beijing China

**Keywords:** lipid transfer protein, cold tolerance, early seedling stage, rice

## Abstract

Nonspecific lipid transfer proteins (nsLTPs) play critical roles in plant development and response to abiotic stresses. Here, we found that a rice lipid transfer protein, OsLTPL159, was associated with cold tolerance at the early seedling stage. Overexpression of an *OsLTPL159* ^IL^ ^112^ allele from the cold‐tolerant introgression line IL112 in either the *japonica* variety Zhonghua17 (ZH17) or the *indica* variety Teqing background dramatically enhanced cold tolerance. In addition, down‐regulation of the expression of *OsLTPL159* in the *japonica* variety ZH17 by RNA interference (RNAi) significantly decreased cold tolerance. Further transcriptomic, physiological and histological analysis showed that the *OsLTPL159* ^IL^ ^112^ allele likely enhanced the cold tolerance of rice at the early seedling stage by decreasing the toxic effect of reactive oxygen species, enhancing cellulose deposition in the cell wall and promoting osmolyte accumulation, thereby maintaining the integrity of the chloroplasts. Notably, overexpression of another allele, *OsLTPL159* ^GC^ ^2^, from the recipient parent Guichao 2 (GC2), an *indica* variety, did not improve cold tolerance, indicating that the variations in the *OsLTPL159* coding region of GC2 might disrupt its function for cold tolerance. Further sequence comparison found that all 22 *japonica* varieties surveyed had an *OsLTPL159* haplotype identical to IL112 and were more cold‐tolerant than the surveyed *indica* varieties, implying that the variations in *OsLTPL159* might be associated with differential cold tolerance of *japonica* and *indica* rice. Therefore, our findings suggest that the *OsLTPL159* allele of *japonica* rice could be used to improve cold tolerance of *indica* rice through a molecular breeding strategy.

## Introduction

Rice (*Oryza sativa* L.) is a staple food for half of the world's population (Sasaki and Burr, 2000). Rice varieties with high and stable yields will be valuable for meeting the challenge of increasing food needs in the future. Rice plants experience abiotic stresses including low and high temperatures (Andaya and Mackill, 2003; Lyman *et al*., 2013; Tian *et al*., 2011), salinity (Ren *et al*., 2005; Wang *et al*., 2017) and drought (Fukao and Xiong, 2013; Uga *et al*., 2013), at both the vegetative and reproductive growth stages. Among these, cold damage poses a major threat to food security in many regions of the world. For example, rice seedlings are sensitive to low temperature in early spring in temperate and subtropical zones as well as in high‐elevation areas, resulting in delayed seedling growth, yellowing, withering, decreased tiller number and stunted growth (Andaya and Mackill, 2003; Tian *et al*., 2011). Especially, in the regions where cultivation technique of direct seeded rice is practised, cold stress is one of the major environmental factors that impairs and delays the early seedling growth of rice, thereby limiting rice productivity. Therefore, improving cold tolerance would be helpful to ensure high and stable yields in rice.

Nonspecific lipid transfer proteins (nsLTPs) can exchange phospholipids between membranes *in vitro* (Kader, 1996; Yeats and Rose, 2008). nsLTPs are small, basic peptides that harbour an N‐terminal hydrophobic signal peptide and eight conserved cysteine residues forming an internal hydrophobic cavity (Kader, 1996). Once the N‐terminal hydrophobic signal peptide is excised, the mature lipid transfer protein (LTP) peptide participates in the cell secretory pathway (Kader, 1996). The nsLTPs are found in the plasma membrane (Debono *et al*., 2009; Edstam *et al*., 2014; Kim *et al*., 2012), cell wall (Thoma *et al*., 1993) and cytoplasm (Guo *et al*., 2013b; Pan *et al*., 2016). Previous studies have shown that *nsLTP* gene products play important roles in many biological processes, including wax assembly (Hollenbach *et al*., 1997), cell wall extension (Nieuwland *et al*., 2005), postmeiotic anther development (Zhang *et al*., 2010), pollen tube tip growth and fertilization (Chae *et al*., 2009), seed development and quality (Wang *et al*., 2015) and pathogen defence responses (Ahmed *et al*., 2017; Gomès *et al*., 2003; Molina and Garcia‐Olmedo, 1997; Park *et al*., 2002; Sarowar *et al*., 2009; Segura *et al*., 1993).

nsLTPs are also involved in plant response to abiotic stresses. For example, the expression level of a probable LTP gene, *TSW12*, increases after NaCl treatment or heat shock in tomato (*Solanum lycopersicum* cv. Rutgers Marglobe; Torres‐Schumann *et al*., 1992). In tree tobacco (*Nicotiana glauca* L. Graham), at least one member of the *NgLTP* gene family is up‐regulated under drought stress, resulting in increased cuticular wax accumulation, which is a nonspecific drought tolerance mechanism (Cameron *et al*., 2006). In *Nicotiana tabacum*, overexpression of *NtLTP4* enhances resistance to salt and drought stresses (Xu *et al*., 2018). In *Arabidopsis thaliana*, overexpression of *AtLTP3* results in increasing survival rates and soluble sugar accumulation as well as reducing electrolyte leakage from chilling stress, which does not significantly affect plant growth and development under normal growth conditions (Guo *et al*., 2013b). Further analysis found that altering the expression level of *AtLTP3* affects plant responses to drought and oxidative stress (Guo *et al*., 2013b). MYB96 (an R2R3‐type MYB transcription factor in *Arabidopsis*) can be directly bound to the promoter region of *AtLTP3 in vivo* and *in vitro* and positively regulates *AtLTP3* (Guo *et al*., 2013b). In maize (*Zea mays*), overexpression of *ZmLTP3*, a homolog of the *Arabidopsis LTP3* gene, also improves tolerance for salt stress (Zou *et al*., 2013). In rice, overexpression of *Oryza sativa DROUGHT‐INDUCED LTP* (*OsDIL*), a gene encoding an LTP, significantly enhances tolerance for drought stress at the vegetative growth stage (Guo *et al*., 2013a). Taken together, these previous studies indicate that the *nsLTP* genes play important roles in responding to abiotic stress in plants. However, the molecular mechanism underlying these effects remains unclear.

In a previous study, we detected seven quantitative trait loci (QTLs) for cold tolerance using an F_2:3_ population derived from a cold‐tolerant introgression line, IL112, and the recipient parent, Guichao 2 (GC2), a cold‐sensitive *indica* variety (Liu *et al*., 2013). Here, we investigated a rice cold‐responsive gene, LOC_Os10g36160, belonging to the *nsLTP* family and named it *OsLTPL159* in the Rice Genome Annotation Project (http://rice.plantbiology.msu.edu/). This gene was located in the mapped region of a cold‐tolerant QTL, *qCST10*. In the cold‐tolerant introgression line IL112, *OsLTPL159* expression was strongly induced by low‐temperature treatment. Further genetic transformation evidence demonstrated that the *OsLTPL159* ^IL112^ allele enhanced rice cold tolerance at the early seedling stage, while the transgenic plants overexpressing a different allele from GC2, *OsLTPL159* ^GC2^, did not show changes in cold tolerance. Sequence analysis showed that the two rice subspecies *O. sativa* L. ssp. *indica* and *O. sativa* L. ssp. *japonica* had different *OsLTPL159* haplotypes, indicating that the variations in *OsLTPL159* might lead to the differential cold tolerance of *japonica* and *indica* rice. Therefore, these findings suggest that the favourable *OsLTPL159* allele of *japonica* rice could be used to improve the cold tolerance of *indica* rice through molecular breeding.

## Results

### *OsLTPL159* encodes a nonspecific lipid transfer protein

In a previous study, we found that the *OsLTPL159* gene (LOC_Os10g36160), encoding a nonspecific LTP, was located in the mapped region of *qCST10*, a QTL for cold tolerance at the early seedling stage (Liu *et al*., 2013). The open reading frame (ORF) of *OsLTPL159* in IL112, a strong cold‐tolerant introgression line, consists of 285 bp and encodes a 94‐amino acid polypeptide. Analysis of the protein sequence using SignalP 4.1 showed that OsLTPL159 has an N‐terminal hydrophobic signal peptide of 25 amino acid residues, which might be required for localization and exact function (Figure S1). The predicted cleavage site is located between the 25th (A) and 26th (Q) amino acid residues, and the cleavage produces a mature protein of 69 amino acid residues (Figure S1) with a predicted molecular mass of 9.90 kDa and an isoelectric point (pI) of 6.07. We constructed a phylogenetic tree based on the amino acid sequence alignment of OsLTPL159 and its homologs in plant species (Figure S2a). Homology analysis showed that homologs of OsLTPL159 exist in various plants, such as *Brachypodium distachyon*,* Sorghum bicolor*,* Zea mays*,* Setaria viridis* and *Arabidopsis thaliana*. Conserved domain analysis showed that OsLTPL159 harbours an eight‐cysteine motif backbone, belonging to a plant nonspecific LTP family (cd01959; Figure S2b). The eight cysteine (C) residues form four disulphide bonds and stabilize a hydrophobic cavity, which allows the binding of different lipids and hydrophobic compounds (Kader, 1996).

### Expression pattern and subcellular localization of OsLTPL159

**Figure 1:**
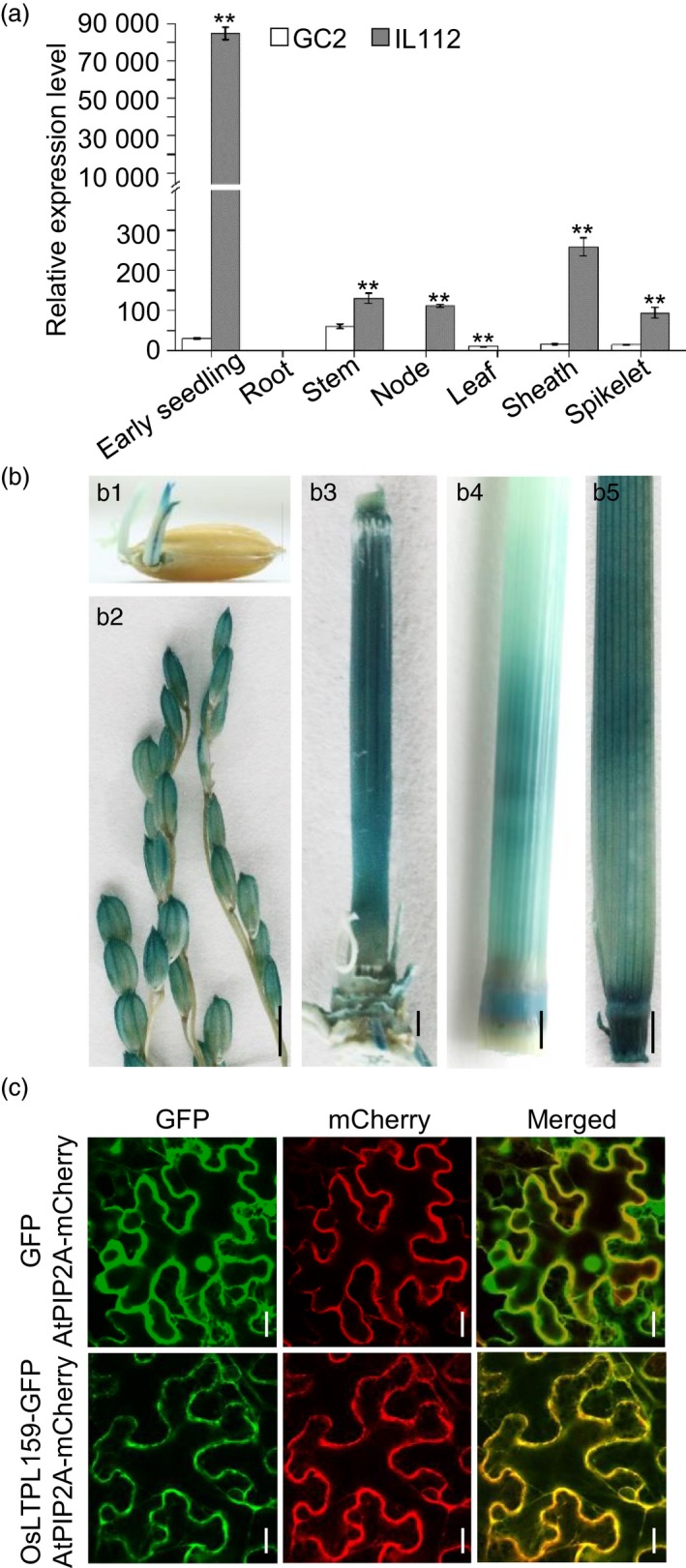
Expression pattern of *OsLTPL159*. (a) Comparison of *OsLTPL159* expression in various tissues of Guichao 2 (GC2) and the introgression line IL112 using RT‐qPCR. Values are means ± SD (*n *=* *3 pooled tissues, 50 plants per pool). Two‐tailed Student's *t*‐tests were conducted to compare GC2 and IL112 (**P <* *0.01). (b) GUS staining of various tissues from the p*O* *sLTPL159* ^IL^ ^112^::*GUS* transgenic plants (T_1_ generation). b1, early seedling; b2, spikelet; b3, stem; b4, stem and node; b5, sheath. Scale bars, 0.5 cm. (c) The OsLTPL159‐GFP fusion protein and the plasma membrane marker AtPIP2A‐mCherry colocalized to the plasma membrane in *Nicotiana benthamiana* epidermal cells. Scale bars, 20 μm.

To determine the expression pattern of *OsLTPL159*, we isolated total RNA from seven tissues of a cold‐tolerant introgression line, IL112, and its recipient parent, Guichao 2 (GC2). Reverse transcription quantitative PCR (RT‐qPCR) analysis indicated that *OsLTPL159* showed higher expression in all surveyed tissues in IL112 than in GC2 and was preferentially expressed in early seedlings, stems, nodes, sheathes and spikelets (Figure 1a). Additionally, we developed a construct, p*OsLTPL159* ^IL112^::*GUS*, harbouring the *GUS* reporter gene under the control of the *OsLTPL159* promoter (1859 bp) from IL112, which was transferred into *japonica* variety Zhonghua17 (ZH17). The results of GUS staining to detect the expression of the resulting fusion protein were consistent with those of the RT‐qPCR analysis (Figure 1b). To detect the subcellular localization of OsLTPL159, we transiently expressed the *OsLTPL159‐GFP* fusion gene under the control of the cauliflower mosaic virus (CaMV) *35S* promoter in *Nicotiana benthamiana* epidermal cells and discovered that the OsLTPL159‐GFP fusion protein colocalized with the plasma membrane marker AtPIP2A‐mCherry (Figure 1c), indicating that the OsLTPL159 protein specifically localized to the plasma membrane.

### *OsLTPL159* is involved in rice cold tolerance at the early seedling stage

**Figure 2:**
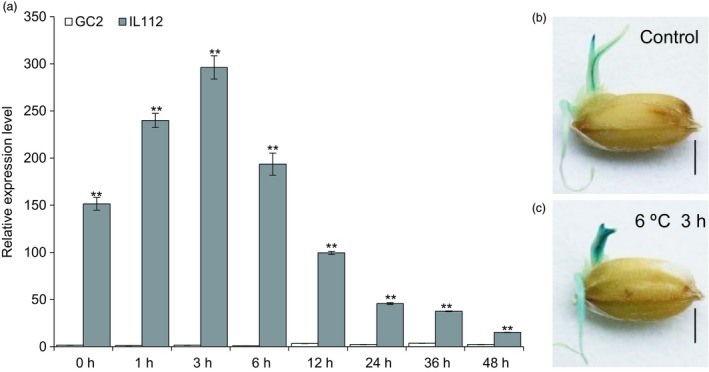
*OsLTPL159* expression was induced by low‐temperature stress. (a) Comparison of *OsLTPL159* expression in GC2 and IL112 under 4 °C treatment. Values are mean ± SD (*n *=* *3 pooled tissues, 50 plants per pool). Two‐tailed Student's *t*‐tests were conducted to compare GC2 and IL112 (**P <* *0.01). (b) and (c) Comparison of GUS activity in early seedlings of p*O* *sLTPL159* ^IL^ ^112^::*GUS* transgenic rice kept in a normal environment (28 °C day/25 °C night temperatures) (b) and at 6 °C for 3 h (c). Scale bars, 0.5 cm.

To validate the cold‐responsiveness of *OsLTPL159* expression, we performed RT‐qPCR to detect the expression level of *OsLTPL159* in rice at the early seedling stage during 48 h of cold treatment. The results showed that in IL112, the expression of *OsLTPL159* was highest after 3 h of cold treatment and then decreased gradually over time, whereas in GC2, *OsLTPL159* was low throughout the course of the cold treatment (Figure 2a). Additionally, we found that the GUS signal in *OsLTPL159* ^IL112^::*GUS* transgenic plants was stronger in the buds and roots of early seedlings after cold treatment than under normal conditions (28 °C day/25 °C night; Figure 2b,c). Further evaluation of the cold tolerance of F_2_ individuals from the GC2 × IL112 cross after cold treatment at 6 °C for 9 days and subsequent recovery at 28 °C for 7 days showed that both heterozygote plants and *OsLTPL159* ^IL112^ homozygote plants had significantly higher average survival rates (measured as the percentage of total seedlings that survived in relation to the total number tested), 75.3% and 80.4%, respectively, than *OsLTPL159* ^GC2^ homozygote plants, which had a survival rate of 34.6% (Figure S3). These results suggested that *OsLTPL159* expression in IL112 was induced by low‐temperature stress, implying that *OsLTPL159* might be involved in cold tolerance of rice at the early seedling stage and that the *OsLTPL159* allele from IL112 was dominant with regard to cold tolerance at the early seedling stage.

**Figure 3:**
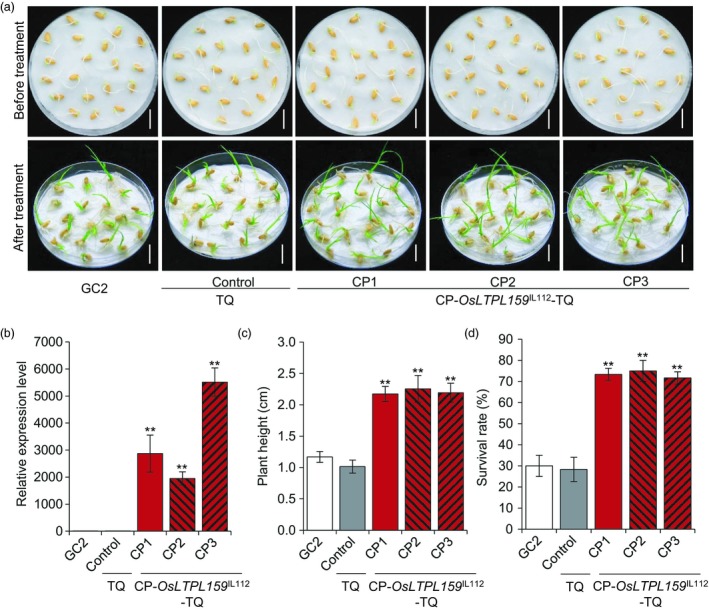
Functional complementation of *OsLTPL159*. (a) Seedling phenotypes of GC2, the control (TQ) and three functional complementation transgenic lines (CP‐*OsLTPL159* ^IL^ ^112^‐TQ) after exposure to 6 °C for 6 day and subsequent recovery at 28 °C for 7 day. Scale bars, 1.5 cm. (b) Comparison of *OsLTPL159* expression in GC2, the control (TQ) and CP‐*OsLTPL159* ^IL^ ^112^‐TQ transgenic lines. Values are means ± SD (*n *=* *3 pooled tissues, 50 plants per pool). (c) and (d) Comparison of plant height (c) and survival rate (d) in GC2, the control (TQ) and CP‐*OsLTPL159* ^IL^ ^112^‐TQ transgenic lines after 6 days at 6 °C and subsequent recovery at 28 °C for 7 days. Values are means ± SD (*n *=* *3 replicates, 20 plants per replicate). Two‐tailed Student's *t*‐tests were conducted to compare the transgenic lines and the controls (**P <* *0.01).

To further investigate whether the *OsLTPL159* ^IL112^ allele in IL112 was associated with cold tolerance, we generated a complementation construct, p*OsLTPL159* ^IL112^, harbouring a 3105‐bp genomic fragment from IL112 that contained the entire *OsLTPL159* ORF with 2586‐bp 5′‐flanking and 234‐bp 3′‐flanking regions. Because GC2 callus tissue has low regeneration ability, we transformed the construct into a different *indica* variety, Teqing (TQ). We obtained 12 independent positive transgenic plants (CP‐*OsLTPL159* ^IL112^‐TQ) and used them to establish transgenic rice lines. Further phenotypic investigation showed that the transgenic CP‐*OsLTPL159* ^IL112^‐TQ lines had significantly greater seedling height (133% more) and survival rates (159% more) than the TQ control plants after cold treatment at 6 °C for 6 days and subsequent recovery at 28 °C for 7 days (Figure 3), indicating that the *OsLTPL159* allele from IL112 played an important role in enhancing cold tolerance at the early seedling stage.

**Figure 4:**
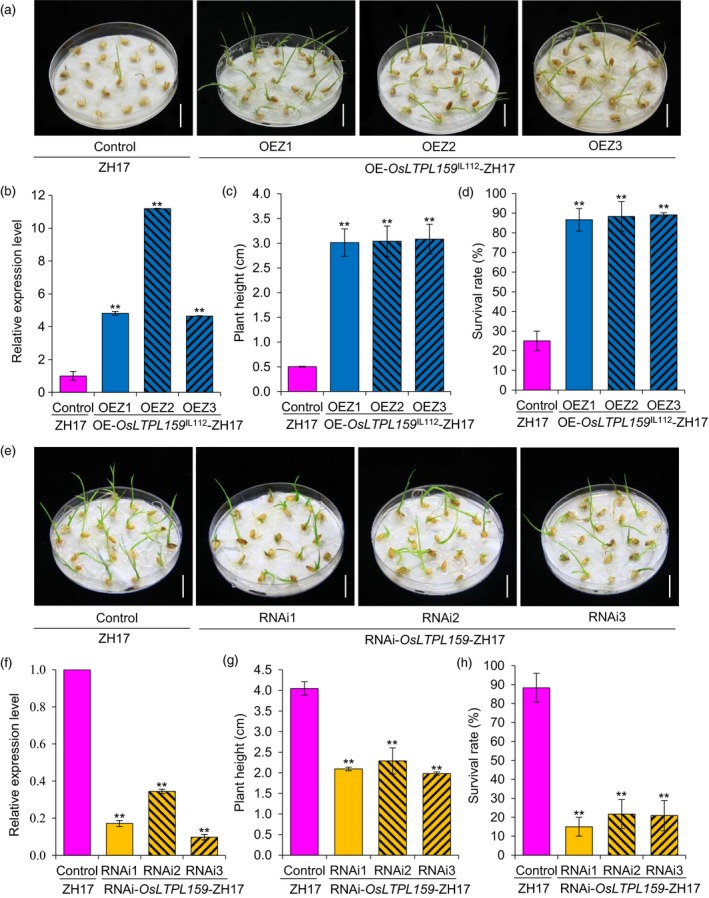
Evaluation of cold tolerance at the early seedling stage in *japonica* variety Zhonghua17 (ZH17) transgenic rice lines overexpressing *OsLTPL159* and in lines expressing an RNAi interference construct of *OsLTPL159*. (a) Phenotypes of the control line (ZH17) and three overexpression transgenic lines (OE‐*OsLTPL159* ^IL^ ^112^‐ZH17) at 2 °C for 9 days and subsequent recovery at 28 °C for 7 days. Scale bars, 1.5 cm. (b) Comparison of *OsLTPL159* expression in the control (ZH17) and overexpression lines (OE‐*OsLTPL159* ^IL^ ^112^‐ZH17). Values are means ± SD (*n *=* *3 pooled tissues, 50 plants per pool). (c) and (d) Comparison of plant height (c) and survival rate (d) in the control (ZH17) and OE‐*OsLTPL159* ^IL^ ^112^‐ZH17 transgenic lines after 9 days at 2 °C and subsequent recovery at 28 °C for 7 days. Values are means ± SD (*n *=* *3 replicates, 20 plants per replicate). (e) Phenotypes of the control (ZH17) and three RNAi transgenic lines (RNAi‐*OsLTPL159*‐ZH17) after 7 days at 2 °C and subsequent recovery at 28 °C for 7 days. Scale bars, 1.5 cm. (f) Comparison of *OsLTPL159* expression in the control (ZH17) and RNAi transgenic lines (RNAi‐*OsLTPL159*‐ZH17). Values are means ± SD (*n *=* *3 pooled tissues, 50 plants per pool). (g) and (h) Comparison of plant height (g) and survival rate (h) in the control (ZH17) and RNAi‐*OsLTPL159*‐ZH17 transgenic lines after 7 days at 2 °C and subsequent recovery at 28 °C for 7 days. Values are means ± SD (*n *=* *3 replicates, 20 plants per replicate). Two‐tailed Student's *t*‐tests were conducted to compare the transgenic lines and the controls (**P <* *0.01).

We also generated an overexpression construct, p*35S*::*OsLTPL159* ^IL112^, and an RNA interference (RNAi) construct, *OsLTPL159*‐RNAi, and separately transferred them into a *japonica* variety, Zhonghua17 (ZH17). RT‐qPCR analysis showed that the expression of *OsLTPL159* was significantly up‐ and down‐regulated in the overexpression and RNAi transgenic plants, respectively, compared to the corresponding nontransformed ZH17 plants. Phenotypic investigation showed that after cold treatment at 2 °C for 9 days and subsequent recovery at 28 °C for 7 days, plants from the *OsLTPL159* overexpression line (OE‐*OsLTPL159* ^IL112^ *‐*ZH17) had higher seedling height (507% more) and survival rate (252% more) than the control plants, while the RNAi transgenic plants (RNAi‐*OsLTPL159*‐ZH17) showed significantly lower seedling height (47% less) and survival rates (78% less) than the control plants (Figure 4). We also transferred the construct p*35S*::*OsLTPL159* ^IL112^ into *indica* variety TQ and found that the overexpression line (OE‐*OsLTPL159* ^IL112^ *‐*TQ) displayed stronger early seedling cold tolerance than the nontransformed TQ control (Figure S4). In addition, investigation of yield‐related traits revealed that none of the traits surveyed, including panicle length, 1000‐grain weight, seed‐setting ratio and grain yield per plant, showed significant differences between the *OsLTPL159* ^IL112^ overexpression lines and their corresponding controls (Figure S5). This suggests that overexpression of *OsLTPL159* ^IL112^ not only significantly increased rice cold tolerance at the early seedling stage but did so without negatively affecting plant growth under normal field conditions.

### Overexpression of *OsLTPL159* protects seedling development against the effects of reactive oxygen species under cold stress

**Figure 5:**
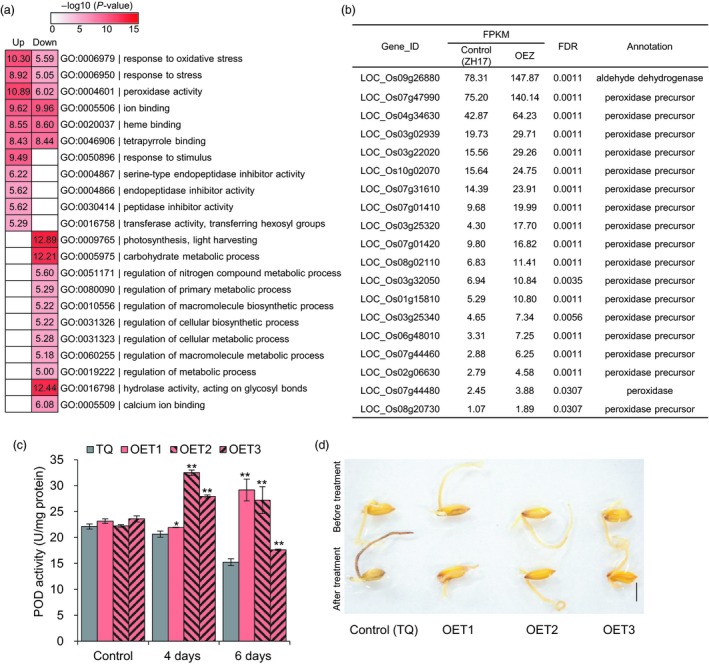
Overexpression of *OsLTPL159* reduces stress damage by minimizing the toxic effects of reactive oxygen species under cold stress. (a) Gene ontology (GO) analysis of 550 up‐regulated and 801 down‐regulated genes in the early seedlings of the overexpression plants OE‐*OsLTPL159* ^IL^ ^112^‐ZH17 (OEZ) and the control plants (ZH17) under cold treatment at 2 °C for 3 h. (b) The expression level of 19 significantly up‐regulated genes involved in oxidative stress. The average FPKM value of all three biological replicates is shown. (c) Comparison of POD activity in the control (TQ) line and overexpression lines OE‐*OsLTPL159* ^IL^ ^112^‐TQ (OET) after 4 and 6 days at 6 °C. Values are means ± SD (*n *=* *3 pooled tissues, 50 plants per pool). Control, before treatment. Two‐tailed Student's *t*‐tests were used to compare the transgenic lines and the corresponding controls (*P <* *0.05, **P <* *0.01). (d) DAB staining of TQ and OE‐*OsLTPL159* ^IL^ ^112^‐TQ transgenic lines before and after cold treatment (6 °C, 6 days). Scale bars, 0.5 cm.

To elucidate the molecular functions of *OsLTPL159* in cold tolerance, we performed RNA‐seq to investigate the genome‐wide gene expression profiles of the overexpression line OE‐*OsLTPL159* ^IL112^‐ZH17 (OEZ) and the control Zhonghua17 (ZH17) under low temperature (2 °C) for 3 h. We identified a total of 1351 differentially expressed genes (DEGs), including 550 up‐regulated and 801 down‐regulated DEGs (fold change ≥1.5, FDR <0.05; Table S1). Gene ontology (GO) analyses revealed that these DEGs were enriched in multiple biological processes, including response to oxidative stress, peroxidase activity, ion binding, heme binding, tetrapyrrole binding and response to stimulus (Figure 5a). After further analysis, we found that a total of 19 genes involved in oxidative stress were significantly up‐regulated in the overexpression plants (Figure 5b). Notably, an up‐regulated gene LOC_Os09g26880 (*OsALDH7*) in the overexpression plants (OEZ) is predicted to encode an aldehyde dehydrogenase involved in reactive oxygen species (ROS) scavenging. This aldehyde dehydrogenase has activities towards malondialdehyde, acetaldehyde and glyceraldehyde, therefore detoxifying the aldehydes generated by lipid peroxidation (Shin *et al*., 2009). These results implied that overexpression of *OsLTPL159* affects the expression of oxidative stress response genes.

To determine whether *OsLTPL159* reduced stress damage by minimizing the toxic effects of ROS, we measured peroxidase (POD) activity of the overexpression plants OE‐*OsLTPL159* ^IL112^‐TQ (OET) and the control TQ plants before and after cold treatment. The results showed that the OET overexpression plants had dramatically higher POD activity after 6 °C treatment for 4 and 6 days, compared with the control plants (Figure 5c). In addition, DAB staining showed that the OET overexpression lines had lower accumulation of H_2_O_2_ than the control plants at 6 °C for 6 days (Figure 5d). Therefore, these findings suggested that overexpression of *OsLTPL159* protects seedling development against the effects of ROS under low‐temperature stress.

### Overexpression of *OsLTPL159* reduces stress damage by enhancing cellulose deposition and promoting osmolyte accumulation

**Figure 6:**
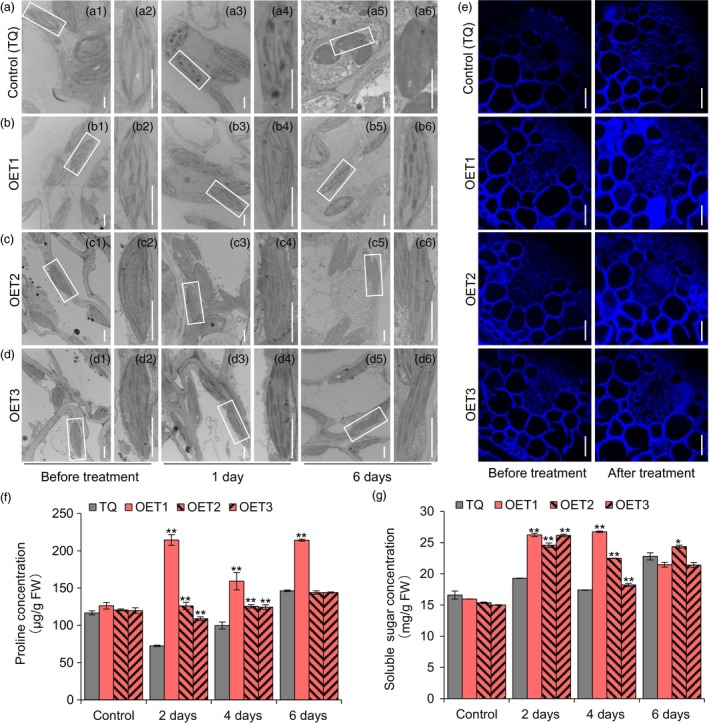
Physiological and histological analysis in the control line (TQ) and the overexpression line OE‐*OsLTPL159* ^IL^ ^112^‐TQ (OET). (a), (b), (c) and (d) Transmission electron microscopy observations of organelle structure in the budburst of control line (TQ) (a) and OE‐*OsLTPL159* ^IL^ ^112^‐TQ transgenic lines (b, c, d) under 6 °C cold treatment. (a1), (b1), (c1) and (d1) Budburst of controls not subjected to cold treatment. The white boxes in (a1), (b1), (c1) and (d1) indicate chloroplasts and are enlarged in (a2), (b2), (c2) and (d2), respectively. (a3), (b3), (c3) and (d3) show the budburst after 1 days at 6 °C. The white boxes in (a3), (b3), (c3) and (d3) indicate the chloroplasts and are enlarged in (a4), (b4), (c4) and (d4), respectively. (a5), (b5), (c5) and (d5) show the budburst after 6 days at 6 °C. The white boxes in (a5), (b5), (c5) and (d5) indicate the chloroplasts and are enlarged in (a6), (b6), (c6) and (d6), respectively. Scale bars, 1 μm. (e) Calcofluor White staining of early seedlings of the control line (TQ) and overexpression lines OE‐*OsLTPL159* ^IL^ ^112^‐TQ before and after cold treatment (6 °C, 6 days). Scale bars, 50 μm. (f) and (g) Comparison of proline (f) and soluble sugar (g) concentration in the control line (TQ) and overexpression transgenic line OE‐*OsLTPL159* ^IL^ ^112^‐TQ after 0–6 days at 6 °C. FW, fresh weight. Control, before treatment. Values are means ± SD (*n *=* *3 pooled tissues, 50 plants per pool). Two‐tailed Student's *t*‐tests were used to compare the transgenic lines and the corresponding controls (*P <* *0.5, **P <* *0.01).

Previous studies reported that cold stress damages stacked grana and disintegrates thylakoid membranes and that cold‐tolerant seedlings restore plasma membrane integrity, thereby protecting chloroplast development under cold stress (Chen *et al*., 2017; Sun *et al*., 2017). To elucidate the physiological mechanism underlying cold tolerance, we compared the cellular morphology of the overexpression plants and control plants TQ using transmission electron microscopy. Under normal conditions, the chloroplasts in both lines had a normal structure with well‐formed lamellae. After 1 day of a 6 °C low‐temperature treatment, the thylakoid membrane networks of some chloroplasts degraded in the TQ plants, while the chloroplasts of the overexpression plants had normal thylakoid grana and thylakoid membranes. After 6 days at 6 °C, the thylakoid membrane networks of all chloroplasts in the control plants broke down and were almost undetectable, whereas the chloroplasts of the overexpression transgenic plants still retained their integral thylakoid membranes (Figure 6a–d). At the same time, we detected cellulose deposition in the cell wall using Calcofluor White M2R staining, which is a nonspecific fluorochrome with the ability to bind with cellulose and chitin (Sumiyoshi *et al*., 2013). The results showed that cellulose deposition was higher in the overexpression plants than in the control plants after 6 days at 6 °C (Figure 6e). In addition, we measured the concentration of osmolytes, including proline and soluble sugars. The results showed that compared with the TQ control, the overexpression plants had higher proline concentration and higher soluble sugar concentration at 2–6 days after low‐temperature treatment (Figure 6f,g). Therefore, these findings suggested that *OsLTPL159* ^IL112^ enhanced cold tolerance of early‐stage rice seedlings by enhancing cellulose deposition and promoting osmolyte accumulation, thereby helping to maintain the integrity of chloroplasts under low‐temperature stress.

### Natural variation in *OsLTPL159* associated with differences in cold tolerance between *japonica* and *indica* subspecies

**Figure 7:**
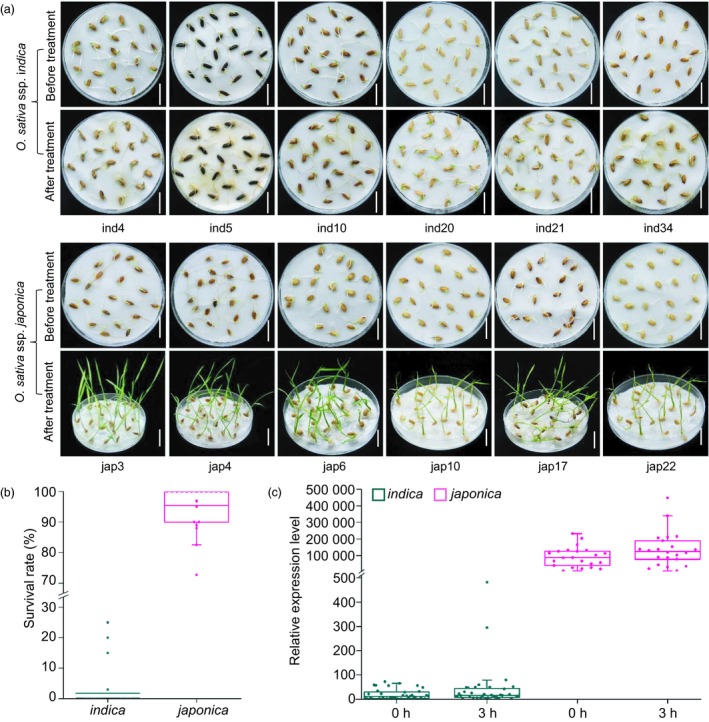
Natural variation in *OsLTPL159* might be involved in the differences in cold tolerance between *japonica* and *indica* subspecies. (a) Phenotypes of several *japonica* and *indica* varieties after treatment for 9 days at 6 °C and subsequent recovery at 28 °C for 7 days. Scale bars, 1.5 cm. (b) Comparison of survival rates in the surveyed *japonica* and *indica* varieties after 9 days at 6 °C and subsequent recovery at 28 °C for 7 days. (c) Comparison of *OsLTPL159* expression in the surveyed *japonica* and *indica* varieties after 0 h or 3 h at 6 °C. Box plot centre and box edges indicate median and 25th or 75th percentiles, respectively, while whiskers indicate the median ± 1.5 × IQR (interquartile range). The dots indicate individual data points. Source data for (b) and (c) are provided in Table S4.

A sequence comparison between IL112 and GC2 revealed 24 single nucleotide polymorphisms (SNPs) and a 15‐bp insertion/deletion (InDel) in the *OsLTPL159* coding region, and 32 SNPs and 16 InDels in the 500‐bp 5′‐flanking region of the gene (Figure S6). In general, *japonica* varieties have stronger cold tolerance than *indica* varieties of rice. Therefore, to investigate whether the variation in the *OsLTPL159* allele was associated with cold tolerance in these two rice subspecies, we next compared the sequences of *OsLTPL159* from 22 *japonica* and 36 *indica* varieties. All surveyed *japonica* varieties had an *OsLTPL159* haplotype identical to that of the introgression line IL112, whereas in all *indica* varieties, the sequences of *OsLTPL159* had high similarity to that of GC2 (Tables S2 and S3). At the same time, we evaluated the cold tolerance of all surveyed varieties at the early seedling stage and found that the 22 *japonica* varieties had significantly stronger cold tolerance than any of the 36 *indica* varieties. Notably, RT‐qPCR analysis showed that the *japonica* varieties had significantly higher expression of *OsLTPL159* than the *indica* varieties under low‐temperature conditions (Figure 7, Table S4), indicating that cold tolerance at the early seedling stage was positively associated with expression level of *OsLTPL159* in rice.

To determine whether the variation in the *OsLTPL159* coding region in *indica* correlated with the difference in cold tolerance, we generated an overexpression construct (OE‐*OsLTPL159* ^GC2^) and transferred it into *indica* variety TQ. Phenotypic investigation showed that the cold tolerance of the *OsLTPL159* ^GC2^ overexpression lines (OE‐*OsLTPL159* ^GC2^‐TQ) did not differ significantly from that of the nontransformed TQ control (Figure S7). Comparison of amino acid sequences revealed that although the OsLTPL159 proteins in both IL112 and GC2 had the same conserved domain consisting of eight cysteine residues creating a stable hydrophobic cavity (Figure S6b), the predicted cleavage site of the N‐terminal hydrophobic signal peptide was located between the 25th (A) and 26th (Q) amino acid residues in IL112, but between the 24th (A) and 25th (V) amino acid residues in GC2 (Figure S1b,c), which might result in OsLTPL159 dysfunction in GC2. Taken together, the variations in *OsLTPL159* seem to alter its expression and function and to be associated with the differential early seedling cold tolerance and environmental adaptation between the *japonica* and *indica* subspecies. Therefore, further identification of existing variations of *OsLTPL159* will be valuable for understanding the molecular mechanisms underlying differential cold tolerance between rice subspecies.

## Discussion

Since the first nsLTP was discovered more than 40 years ago (Kader, 1975), numerous reports related to nsLTPs have been published, mostly covering biochemical aspects of nsLTP proteins, such as their structure, ligand binding and regulation (Salminen *et al*., 2016). Plant nsLTPs are a type of small, ubiquitous and secreted protein (Kader, 1996) and are abundantly present in higher plants. They contain four disulphide bridges formed from eight conserved cysteine residues for stabilizing the hydrophobic cavity (Gomar *et al*., 1996; Heinemann *et al*., 1996; Lee *et al*., 1998; Maldonado *et al*., 2002; Shin *et al*., 1995) and two consensus pentapeptide motifs (Douliez *et al*., 2000). In recent years, more results have supported the theory that *nsLTPs* are associated with resistance to abiotic and biotic stress in plants. Based on previous studies, *nsLTP* genes are responsive to various stresses, including salt (Safi *et al*., 2015; Zou *et al*., 2013), drought (Guo *et al*., 2013a), abscisic acid (ABA; Arondel *et al*., 2000; Yubero‐Serrano *et al*., 2003), heat (Wang *et al*., 2014) and cold treatment (Gangadhar *et al*., 2016; Hincha *et al*., 2001; Yubero‐Serrano *et al*., 2003). For example, in *Arabidopsis*, the *LTP* genes *DEFECTIVE IN RESISTANCE1* (*DIR1*) (Maldonado *et al*., 2002), *LIPID TRANSFER PROTEIN3* (*LTP3*) (Guo *et al*., 2013b) and *AZELAIC ACID INDUCED1* (*AZI1*) (Pitzschke *et al*., 2014) are involved in systemic resistance signalling, drought and freezing tolerance, and salt tolerance, respectively, and other *nsLTPs* have been associated with clubroot disease (Jülke and Ludwig‐Müller, 2015). Here, we characterized a rice *LTP* gene, *OsLTPL159*, from the introgression line IL112 and explored its function in cold tolerance. Overexpression of an *OsLTPL159* ^IL112^ allele from IL112 in either the *japonica* variety ZH17 or the *indica* variety TQ background dramatically enhanced cold tolerance at the early seedling stage. By contrast, down‐regulation of *OsLTPL159* expression in *japonica* ZH17 significantly decreased cold tolerance at the early seedling stage.

Plants respond to abiotic stresses with an increase in ROS, which have toxic effects on plant development by damaging DNA, lipids, proteins and other macromolecules. Plants maintain normal development under abiotic stresses by balancing the rate of ROS production and ROS scavenging. In this study, we found the DEGs between the *OsLTPL159* overexpression plants and the control plants were enriched in response to oxidative stress and peroxidase activities. Compared with the control plants, the overexpression plants had dramatically higher POD activity and lower accumulation of H_2_O_2_ under cold stress. Hence, these results suggested that overexpression of *OsLTPL159* protects seedling development by maintaining ROS homoeostasis, thus avoiding the deleterious effects of ROS under low‐temperature stress. Notably, abiotic stress can cause ROS accumulation in the cell wall, thereby remodelling the wall (Tenhaken, 2015). In this study, we found that the *OsLTPL159* overexpression plants had higher cellulose deposition in the cell wall than the control plants under cold stress. Meanwhile, overexpression of *OsLTPL159* also promoted osmolyte accumulation and maintained the integrity of chloroplasts. Therefore, these results indicated that cold tolerance in the *OsLTPL159* overexpression plants was achieved by several mechanisms acting in an orchestrated manner.

Cold tolerance is a complex trait in plants. In rice, many genes and QTLs associated with cold stress have been identified, providing valuable genetic resources for enhancing cold tolerance through molecular breeding (Andaya and Mackill, 2003; Fujino *et al*., 2008; Liu *et al*., 2018a,b; Lu *et al*., 2014; Ma *et al*., 2015; Mao *et al*., 2019; Saito *et al*., 2010; Zhang *et al*., 2017; Zhao *et al*., 2016, 2017). The QTL *qLTG3‐1* controls low‐temperature germinability, and a 71‐bp deletion in the coding region of *qLTG3‐1* causes a decrease in low‐temperature germinability in rice variety Hayamasari (Fujino *et al*., 2008). The QTL *COLD1*, which encodes a regulator of G‐protein signalling, confers chilling tolerance in *japonica* rice. Overexpression of the *japonica* allele *COLD1* ^*jap*^ could significantly enhance chilling tolerance of rice seedlings (Ma *et al*., 2015). The QTL *qCTS‐9* for cold resistance at the rice seedling stage was identified by combining QTL mapping and expression profiling analysis, and overexpression of *qCTS‐9* can enhance cold tolerance at the seedling stage by reducing ion permeability (Zhao *et al*., 2017). The major QTL for rice seedling cold tolerance, *qCTS12*, was narrowed down to an approximately 87‐kb region on chromosome 12 (Andaya and Tai, 2006), and naturally occurring OsGSTZ2 isoforms in the *qCTS12* fine‐mapped region were associated with the differential response to low‐temperature stress in rice *indica* and *japonica* subspecies at the seedling stage (Kim *et al*., 2011). A functional nucleotide polymorphism in the promoter of *HAN1* is associated with the chilling tolerance of temperate *japonica* rice and is believed to have allowed rice to adapt to a temperate climate during its northward expansion (Mao *et al*., 2019). A bZIP transcription factor encoded by *bZIP73* participates in the adaptation to cold climates in *japonica* subspecies (Liu *et al*., 2018a). *Ctb1*, encoding an F‐box protein, interacts with a subunit of the E3 ubiquitin ligase, Skp1, suggesting that a ubiquitin–proteasome pathway is involved in cold tolerance at the booting stage (Saito *et al*., 2010). A single amino acid substitution (I357K) in *LTG1*, encoding a casein kinase I, is related to growth rate, heading period and yield of rice under cold stress (Lu *et al*., 2014). Natural variations in the QTL *CTB4a*, encoding a conserved leucine‐rich repeat receptor‐like kinase, could enhance rice adaptation to cold habitats at the booting stage (Zhang *et al*., 2017). In the present study, genetic evidence demonstrated that the variations found in *OsLTPL159* cause the differential cold tolerance between IL112 and its cold‐sensitive parental line GC2. In addition, we found that the natural variations in *OsLTPL159* were associated with the differential cold tolerance and environmental adaptation in rice *indica* and *japonica* subspecies at the early seedling stage. Further investigation of the key variations in OsLTPL159 that affect its transcription and function could provide new insights into the molecular mechanism underlying cold tolerance and environmental adaptation in rice at the early seedling stage. Notably, phenotypic evaluation revealed that overexpression of *OsLTPL159* ^IL112^ not only significantly enhanced rice cold tolerance at the early seedling stage, but did so without penalty to the yield of rice under normal field conditions. Hence, the favourable allele of *OsLTPL159* from *japonica* rice could be used in the future to directly improve the cold tolerance of *indica* rice through molecular breeding.

## Experimental procedures

### Plant materials

IL112, a strong cold‐tolerant line, was identified in the introgression line derived from an advanced backcross between Dongxiang common wild rice (*Oryza rufipogon* Griff.), as donor parent, and an *indica* variety Guichao 2 (GC2), as recipient parent (Liu *et al*., 2013; Tian *et al*., 2006). An F_2_ population derived from the cross between IL112 and GC2 was genotyped using an insertion/deletion marker (*OsLTPL159*‐indel), and cold tolerance was evaluated at the early seedling stage (the buds with approximately 5 mm in length) with five replicates (36 individuals per replicate). For sequence and expression analysis of *OsLTPL159* and cold tolerance evaluation, a total of 22 *japonica* and 36 *indica* varieties were used (listed in Table S4).

### Low‐temperature treatment

Seeds were incubated at 42 °C for approximately 2 days to break dormancy and soaked in deionized water at 37 °C for about 2 days for germination. Germinated seeds were transferred to a Petri dish with two layers of filter paper in a greenhouse (28 °C day/25 °C night temperatures, 12‐h/12‐h light/dark cycle and 85% relative humidity). Early seedlings (the buds with approximately 5 mm in length) were subjected to low‐temperature treatment (2–6 °C) for 6–9 days in incubators and subsequent recovery at 28 °C for 7 days. Finally, the plant height and survival rate (measured as the percentage of total seedlings that survived in relation to the total number tested) of 20 plants per replicate were measured to evaluate cold tolerance with three replicates.

### PCR and RT‐qPCR analyses

After cold treatment, both buds and roots were harvested, frozen in liquid nitrogen and stored at −80 °C for total RNA extraction. The control plants were also harvested and stored under the same conditions. Total RNA was extracted using TRIzol reagent (Invitrogen, Carlsbad, CA) and purified using a Qiagen RNeasy Kit (Qiagen, Hilden, Nordrhein‐Westfalen, Germany). First‐strand cDNAs were synthesized using a SuperScript III RT Kit (Invitrogen). The RT‐qPCRs were performed in a CFX96 Real‐Time System (Bio‐Rad, Hercules, CA) with the rice *UBIQUITIN* gene (LOC_Os03g13170; Jiang *et al*., 2019) as the internal control under the following conditions: initial denaturation at 95 °C for 3 min and then 40 cycles of 95 °C for 15 s, 58 °C for 30 s and 72 °C for 30 s. LinRegPCR was used to analyse the PCR product curves to determine the efficiency of the exponential section of the product curve by linear regression (Ramakers *et al*., 2003). The relative expression levels were calculated from three replicate RT‐qPCR experiments (Schmittgen and Livak, 2008).

### Generation of constructs and genetic transformation

A 3181‐bp genomic fragment from IL112, harbouring the *OsLTPL159* coding region with 2586‐bp 5′‐flanking and 234‐bp 3′‐flanking regions, was inserted into the binary vector p*CAMBIA1300* to form the functional complementation construct p*OsLTPL159* ^IL112^. The constructs p35S::*OsLTPL159* ^IL112^ and p35S:*OsLTPL159* ^GC2^ harboured the *OsLTPL159* ORF from IL112 or Guichao 2 (GC2), respectively, under the control of the CaMV *35S* promoter. The RNAi construct was generated by cloning the sense and antisense fragments of *OsLTPL159* into the pTCK303 vector, creating a fusion with the maize *Ubiquitin* promoter (Wang *et al*., 2004). All plasmid constructs were introduced into *Agrobacterium tumefaciens* strain EHA105 and then transferred into *indica* variety Teqing (TQ) and/or *japonica* variety Zhonghua17 (ZH17) (Jiang *et al*., 2019).

### GUS staining

To investigate the expression pattern of *OsLTPL159*, we generated a construct (p*OsLTPL159* ^IL112^::*GUS*) in which the *OsLTPL159* promoter of IL112, harbouring the entire 1.8‐kb region upstream from the start codon of *OsLTPL159*, was fused with the *GUS* reporter gene, and then cloned the result into the binary vector p*CAMBIA1300*. The p*OsLTPL159*::*GUS* construct was introduced into the *A. tumefaciens* strain EHA105 and then transferred into *japonica* variety Zhonghua17 (ZH17). GUS staining of tissues from the positive transgenic plants was performed as previously described (Fujino *et al*., 2008).

### Subcellular localization

To investigate the subcellular localization of OsLTPL159, we made a construct (p*35S*::*OsLTPL159*‐*GFP*) in which the *OsLTPL159* coding region fused with *GFP* was driven by the CaMV *35S* promoter. The plasmid p*35S*::*OsLTPL159*‐*GFP* and the plasma membrane marker AtPIP2A (plasma membrane intrinsic protein 2A, At3 g53420)‐mCherry (Cutler *et al*., 2000) were introduced into the *A. tumefaciens* strain EHA105 and then transfected into *N. benthamiana* epidermal cells. The infected tissues were observed with a confocal laser‐scanning microscope (Olympus FV1000).

### RNA‐seq analysis

Total RNA was isolated from early‐stage seedlings (the buds with approximately 5 mm in length) of the overexpression plants OE‐*OsLTPL159* ^IL112^‐ZH17 (OEZ) and the control plants (ZH17) under low‐temperature treatment at 2 °C for 3 h, with three biological replicates each containing 50 plants. Paired‐end libraries were constructed and sequenced using an Illumina HiSeq2500 at the Novogene company (China). The raw reads were mapped to the reference genome (Os‐Nipponbare‐Reference‐IRGSP‐1.0, MSU7) using TopHat2 with the default parameters (Kim *et al*., 2013). Cuffdiff was used to calculate the FPKM (fragments per kilobase of exon per million mapped reads) of each gene and to identify the DEGs (fold change ≥1.5, FDR <0.05). GO enrichment analysis was performed using agriGOv2 (Tian *et al*., 2017).

### Transmission electron microscopy

For the transmission electron microscope assay, detached buds were fixed in fixation buffer (2.5% glutaraldehyde in phosphate buffer, pH 7.2) for 12 h at 4 °C. The plant tissues were rinsed and post‐fixed in a secondary fixation buffer (1% OsO_4_, w/v) at 4 °C, dehydrated through an ethanol series and embedded in Spurr's medium (Tanaka *et al*., 2003). Ultrathin sections were double‐stained with uranyl acetate and lead citrate aqueous solution and then observed using a JEM‐1230 transmission electron microscope (JEOL, Japan) at 80 kV.

### Calcofluor White staining

For analysing the cellulose deposition in cell walls of the early seedlings, detached budbursts were sliced using a cryostat microtome (Leica CM3050S). The sections were stained with 1 g/L Calcofluor White M2R (Sigma, St. Louis, MO) and observed with a confocal laser‐scanning microscope (Olympus FV1000).

### Free proline and soluble sugars concentration

Free proline concentration was measured as described previously (Liu *et al*., 2018b). Approximately 200 mg early seedling tissue was homogenized in 2 mL sulphosalicylic acid (3%), followed by centrifugation at 13 000 ***g*** for 15 min at 4 °C. The extract (0.5 mL) was transferred to a new microcentrifuge tube and mixed with 1 mL acid ninhydrin and 1 mL acetic acid. The reaction mixture was boiled in a water bath at 100 °C for 30 min, cooled at 4 °C for 30 min and thoroughly mixed with 1 mL toluene. Finally, the absorbance of 0.2 mL of the toluene phase (upper phase) was read at 520 nm using a spectrophotometer (PowerWave XS2). Soluble sugar concentration was measured following the methods used previously (Wang *et al*., 2018) with minor modifications. The early seedling tissues (0.2 g) were ground with 1 mL of distilled water, heated at 100 °C for 10 min, cooled, centrifuged at 8000 ***g*** for 10 min at 25 °C and diluted to a volume of 10 mL with distilled water. The 0.3 mL reaction mixture contained 0.04 mL of the extract, 0.04 mL of distilled water, 0.02 mL of the mixed reagent (1 g anthrone and 50 mL ethyl acetate) and 0.2 mL of 98% (w/v) H_2_SO_4_. The control mixture contained 0.04 mL of distilled water instead of the extract. The mixture was heated at 95 °C for 10 min, and the absorbance was determined at 620 nm using a microplate reader (PowerWave XS2). Soluble sugar concentration was calculated using glucose as the standard. Three biological replicates were analysed for each treatment.

### POD activity and DAB stain

Measurement of POD activity was performed as previously described (Huang *et al*., 2013). To detect H_2_O_2_ accumulation, the early seedlings were placed in 1 mg/mL 3,3′‐diaminobenzidine (DAB)–HCl solution (pH 3.8) and incubated in the dark for 8 h; then, the chlorophyll was cleared in 80% (v/v) ethanol solution at room temperature for 2 days (Faivre‐Rampant *et al*., 2008).

### Phylogenetic analysis

All protein sequences were searched using BLASTP in the GenBank protein database (http://www.ncbi.nlm.nih.gov/BLAST/) with the OsLTPL159 protein sequence as a query, and the results were inspected manually. Sequences were aligned using CLUSTAL_X followed by manual alignment (Thompson *et al*., 1997). Phylogenetic trees were constructed based on the neighbour‐joining algorithm with the option of pairwise deletion using MEGA 7.0 (Kumar *et al*., 2016). Bootstraps with 1000 replicates were estimated to test inferred phylogeny. Motif analyses were conducted using WebLogo (http://weblogo.berkeley.edu/logo.cgi).

### Bioinformatic analysis

The cleavage sites and signal peptide in the OsLTPL159 protein were predicted using SignalP 4.1 (http://www.cbs.dtu.dk/services/SignalP/; Petersen *et al*., 2011). The theoretical calculation of molecular weight was determined with ExPASy using the Compute pI/Mw tool (http://www.expasy.ch/tools/pi_tool.html).

### Statistical analysis

A two‐tailed Student's *t*‐test was used to compare data from two groups, and Tukey's honestly significant difference analysis was used to compare multiple groups, using SPSS version 16 (SPSS Inc, Chicago).

### Primers

The primers used in this study are listed in Table S5.

### Accession numbers

The RNA‐seq data derived from the *OsLTPL159* overexpression plants (OEZ) and the control plants (ZH17) have been deposited in NCBI's Gene Expression Omnibus under accession number GSE131083.

## Conflict of interest

The authors declare no conflict of interest.

## Author contributions

F.L. conceived and designed the experiments. J.Z. performed most of the experiments. S.W., J.Q. and C.S. provided technical assistance and conducted the collection and maintenance of rice germplasm. J.Z. and F.L. performed data analysis and wrote the manuscript.

## Supporting information


**Figure S1** Sequence analysis of *OsLTPL159*. (a) Full‐length cDNA of *OsLTPL159* in IL112 and the deduced amino acid sequence.
**Figure S2** Phylogenetic and conserved domain analysis of OsLTPL159 and its homologs in plants.
**Figure S3** Comparison of cold tolerance at the early seedling stage in Guichao 2 (GC2), IL112, and F_2_ individuals from a GC2 × IL112 cross.
**Figure S4** Evaluation of cold tolerance at the early seedling stage in *indica* variety Teqing (TQ) transgenic lines overexpressing *OsLTPL159*.
**Figure S5** Evaluation of yield‐related traits in the overexpression transgenic lines in the field.
**Figure S6** Sequence comparison of *OsLTPL159* between Guichao 2 (GC2) and IL112.
**Figure S7** Evaluation of cold tolerance at the early seedling stage in transgenic lines overexpressing *OsLTPL159* ^GC2^ from *indica* variety Guichao 2.

**Table S1** Differentially expressed genes between the control plants (ZH17) and the overexpression plants OE‐*OsLTPL159* ^IL112^‐ZH17 (OEZ) under low‐temperature stress (2 °C, 3 h) detected using RNA‐seq.
**Table S2** Comparison of the 24 SNPs and one insertion/deletion (InDel) in the coding region of *OsLTPL159* from GC2, IL112, 36 *indica* varieties and 22 *japonica* varieties.
**Table S3** Comparison of the 32 SNPs and 16 insertions/deletions (InDels) in the 500‐bp 5′‐flanking region of *OsLTPL159* from GC2, IL112, 36 *indica* varieties, and 22 *japonica* varieties.
**Table S4** The survival rate at the early seedling stage and the expression level of *OsLTPL159* in 36 *indica* varieties and 22 *japonica* varieties.
**Table S5** Primers used in this study.
