# Supplementary material for: The lipid transfer protein OsLTPL159 is involved in cold tolerance at the early seedling stage in rice
Source: Plant Biotechnol J. 2019 Sep 11;18(3):756–69. doi: 10.1111/pbi.13243 (PMC7004919; doi:10.1111/pbi.13243)
Supplement: Supplementary file 1 — Figure S1 Sequence analysis of OsLTPL159. (a) Full‐length cDNA of OsLTPL159 in IL112 and the deduced amino acid sequence. Figure S2 Phylogenetic and conserved domain analysis of OsLTPL159 and its homologs in plants. Figure S3 Comparison of cold tolerance at the early seedling stage in Guichao 2 (GC2), IL112, and F2 individuals from a GC2 × IL112 cross. Figure S4 Evaluation of cold tolerance at the early seedling stage in indica variety Teqing (TQ) transgenic lines overexpressing OsLTPL159. Figure S5 Evaluation of yield‐related traits in the overexpression transgenic lines in the field. Figure S6 Sequence comparison of OsLTPL159 between Guichao 2 (GC2) and IL112. Figure S7 Evaluation of cold tolerance at the early seedling stage in transgenic lines overexpressing OsLTPL159 GC2 from indica variety Guichao 2. [file PBI-18-756-s001.pdf]

(a)

1 acaccaccacatcgataacattcgatcacattcggctagtaaccaagcagttcttacgct
61 agagctagctcgagcaatggtgaagtgggcagctgtgatggagatgcttctgctgacggc
M V K W A A V M E M L L L T A
121 agcagcgacggcggtagcggtggtggcggcgcagtgtgaccctgagcagctatcggcgtg
A A T A V A V V A A Q C D P E Q L S A C
181 cgtgagcccgatcttctacgggacggcgccatcagagtcgtgctgctccaacctacgcgc
V S P I F Y G T A P S E S C C S N L R A
241 acagcagaaggaggggtgcctctgccagtacgcgaaagacccgacgtacgcgtcctacgt
Q Q K E G C L C Q Y A K D P T Y A S Y V
301 caacaacaccaacgcacgcaagaccatcgccgcctgcggcatccccattcccagctgcta
N N T N A R K T I A A C G I P I P S C *
361 ggctcgatctcccgccgccgccggccatgatgcgtacgtggcatatatacgcgtggtgtg
421 tacgtgcgcttgaagaaaagggcgtgagtttgaattaataattatccagtacgcgatgtt
481 tgatcgatcgatgatgtgcctttttttttggtatttgaagtttgaactctgaatttcat
541 cttgtgttaattggagtatgatagctgtgtgtacattcgttttggaggtagaggccggtt
601 taattca

(b)

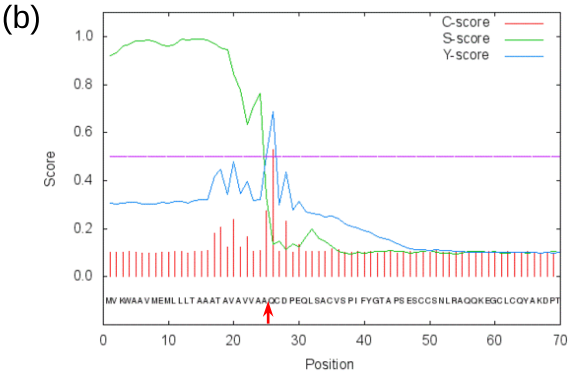

(c)

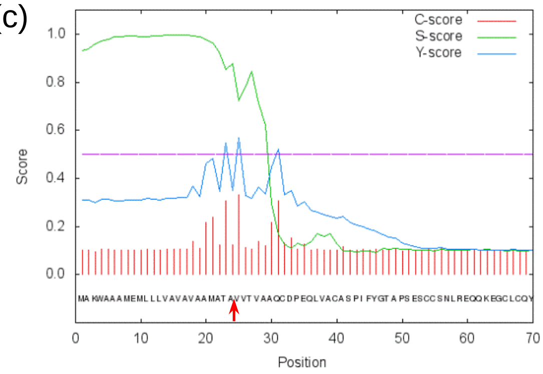

Figure S1. Sequence analysis of *OsLTPL159*. (a) Full-length cDNA of *OsLTPL159* in IL112 and the deduced amino acid sequence. Lowercase letters and uppercase letters indicate the nucleotide and amino acid sequence, respectively. The box indicates the signal peptide at the N terminus. Red letters indicate the eight conserved cysteine (C) residues. Asterisk indicates termination codon. (b) and (c) Predicted signal peptide of OsLTPL159 in IL112 (b) and Guichao 2 (GC2) (c) using SignalP4.1 (http://www.cbs.dtu.dk/services/SignalP). The red arrows indicate the predicted cleavage site.

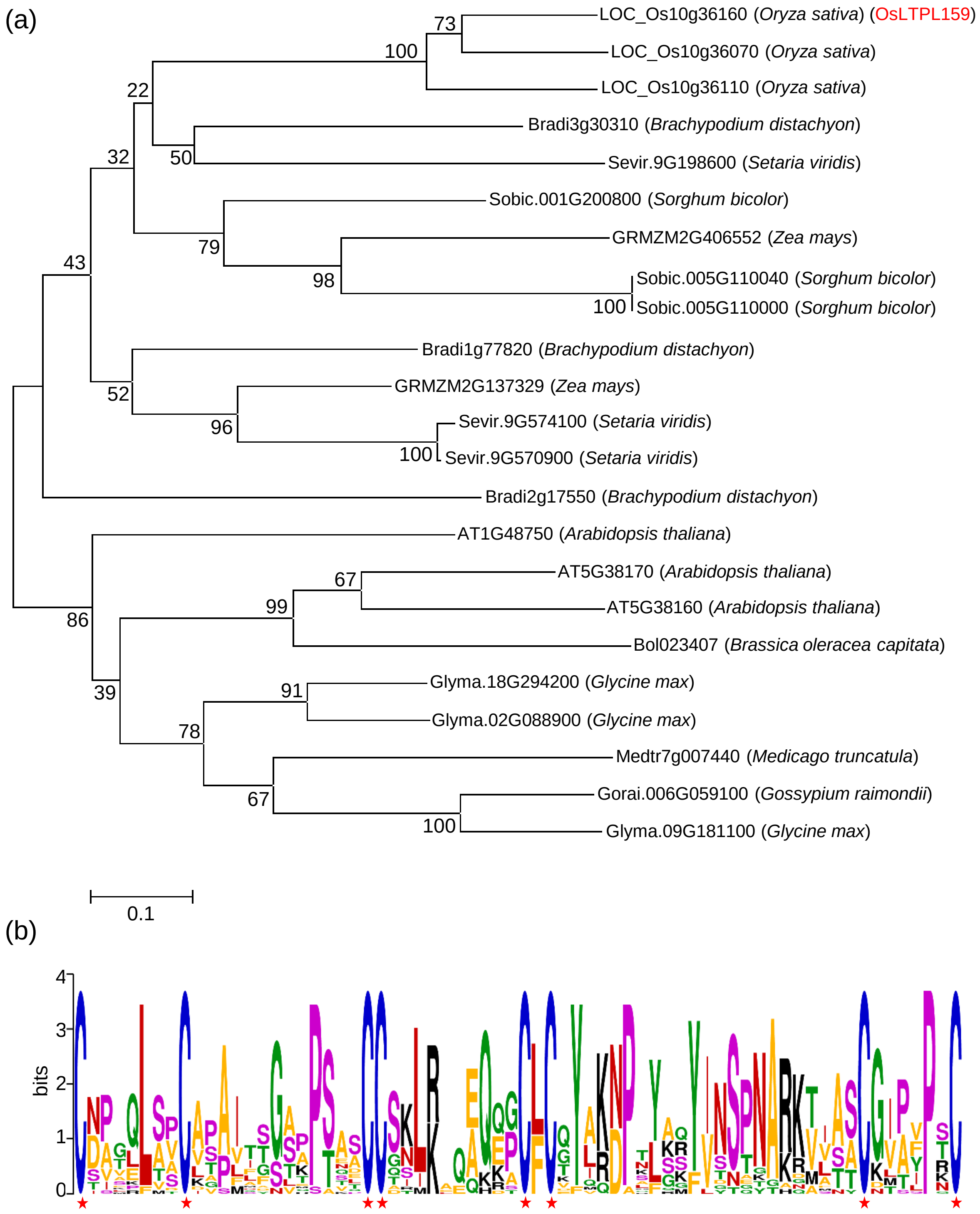

Figure S2. Phylogenetic and conserved domain analysis of OsLTPL159 and its homologs in plants. (a) Phylogenetic analysis of OsLTPL159 and its homologs in plants. The neighbor-joining tree was constructed using MEGA 7.0 with default parameters. Numbers indicate bootstrap support based on 1,000 replicates. Branch length indicates substitutions per site. (b) Conserved domain analysis of OsLTPL159 and its homologs in plants. The consensus sequence depicts amino acid sites, the height of the symbols indicates the relative frequency of each amino acid at that position, and red stars represent the eight cysteine (C) residues in the non-specific lipid transfer proteins.

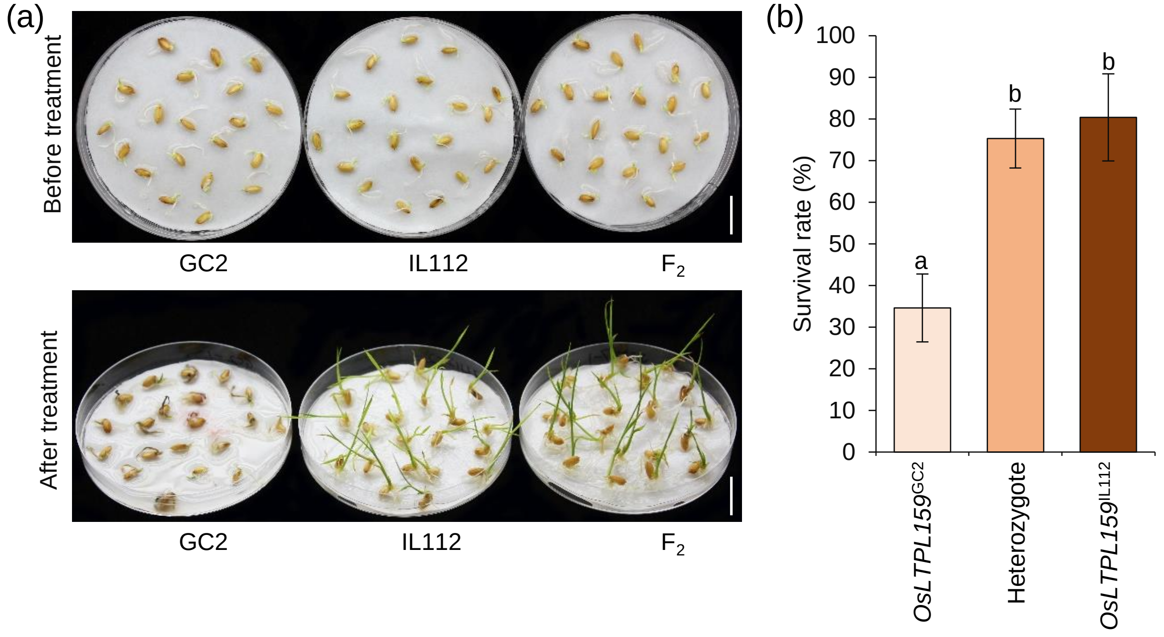

Figure S3. Comparison of cold tolerance at the early seedling stage in Guichao 2 (GC2), IL112, and F_2 individuals from a GC2 × IL112 cross. (a) Phenotypes of GC2, IL112, and F_2 individuals after treatment at 6 °C for 9 d and subsequent recovery at 28 °C for 7 d. Scale bars, 1.5 cm. (b) Comparison of survival rates in *OsLTPL159*^{GC2} homozygote, heterozygote, and *OsLTPL159*^{IL112} homozygote plants from a GC2 × IL112 cross after treatment at 6 °C for 9 d and subsequent recovery at 28 °C for 7 d. Values are expressed as mean ± SD (n = 5 replicates, 36 plants per replicate). Different letters denote significant differences ($P < 0.01$) determined using Tukey's honestly significant difference analysis.

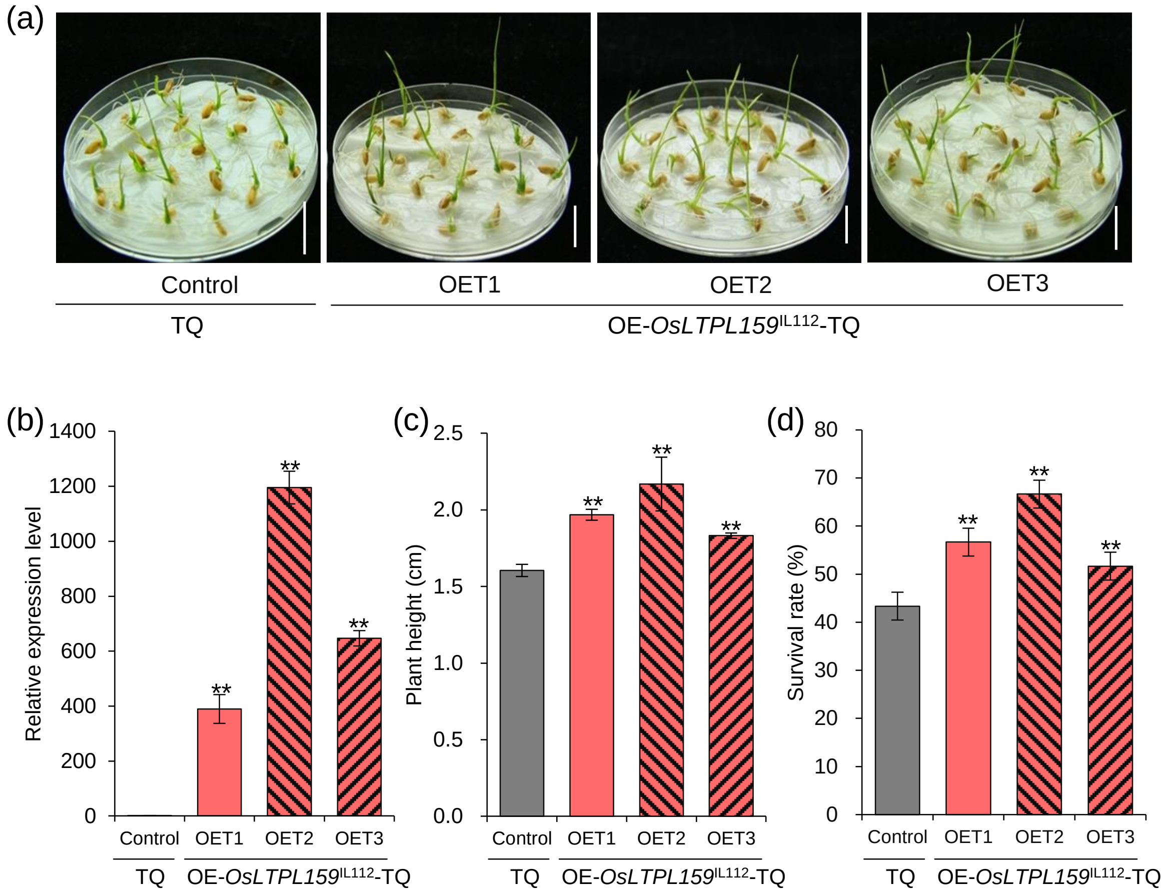

Figure S4. Evaluation of cold tolerance at the early seedling stage in *indica* variety Teqing (TQ) transgenic lines overexpressing *OsLTPL159*. (a) Phenotypes of the control line (TQ) and three overexpression lines (OE-*OsLTPL159*^{IL112}-TQ) after 6 d at 6 °C and a subsequent 7 d of recovery at 28 °C. Scale bars, 1.5 cm. (b) Comparison of *OsLTPL159* expression in the control (TQ) and OE-*OsLTPL159*^{IL112}-TQ transgenic lines. Values are means ± SD (n = 3 pooled tissues, 50 plants per pool). (c) and (d) Comparison of plant height (c) and survival rate (d) in the control (TQ) and OE-*OsLTPL159*^{IL112}-TQ transgenic lines after 6 d at 6 °C and a subsequent 7 d of recovery at 28 °C. Values are means ± SD (n = 3 replicates, 20 plants per replicate). Two-tailed Student's *t*-tests were conducted to compare the transgenic lines and the controls (**$P < 0.01$).

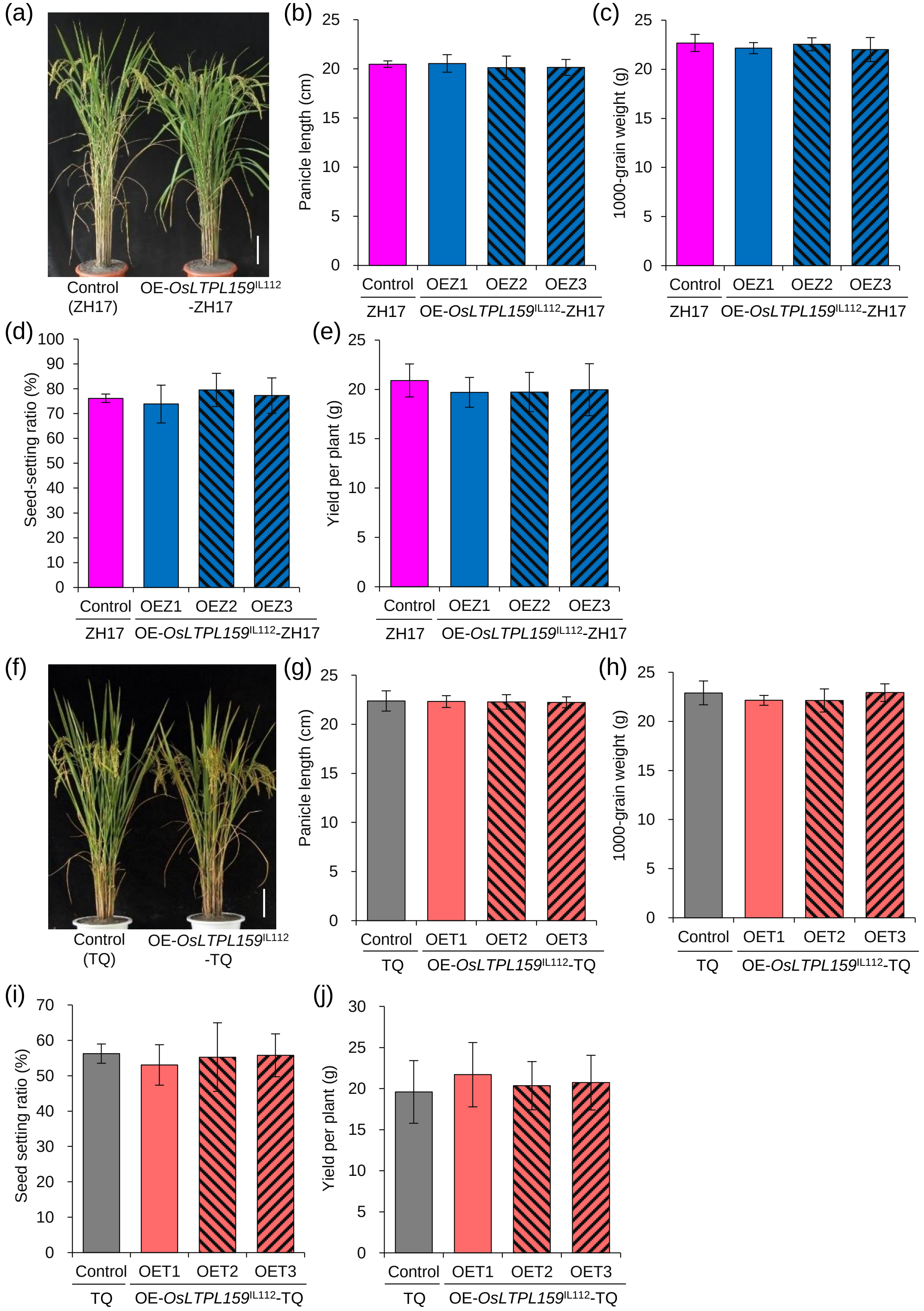

(a)
Control
(ZH17)
OE-OsLTPL159IL112
-ZH17
(b)
Panicle length (cm)
Control
OEZ1
OEZ2
OEZ3
ZH17
OE-OsLTPL159IL112-ZH17
(c)
1000-grain weight (g)
(d)
Seed-setting ratio (%)
(e)
Yield per plant (g)
(f)
Control
(TQ)
OE-OsLTPL159IL112
-TQ
(g)
Panicle length (cm)
OET1
OET2
OET3
TQ
OE-OsLTPL159IL112-TQ
(h)
1000-grain weight (g)
(i)
Seed setting ratio (%)
(j)
Yield per plant (g)

Figure S5. Evaluation of yield-related traits in the overexpression transgenic lines in the field. (a) Phenotypes of the control line (ZH17) and overexpression line OE-*OsLTPL159*^{IL112}-ZH17 at the mature stage. Scale bars, 8.5 cm. (b)–(e) Comparison of yield-related traits, including panicle length (b), 1000-grain weight (c), seed setting ratio (d), and grain yield per plant (e), in the control (ZH17) and overexpression transgenic line OE-*OsLTPL159*^{IL112}-ZH17. (f) Phenotypes of the control line (TQ) and overexpression transgenic line OE-*OsLTPL159*^{IL112}-TQ at the mature stage. Scale bars, 8.5 cm. (g)–(e) Comparison of yield-related traits, including panicle length (g), 1000-grain weight (h), seed setting ratio (i), and grain yield per plant (j), in the control (TQ) and overexpression transgenic line OE-*OsLTPL159*^{IL112}-TQ. Values are means $\pm$ SD (n = 15 plants). Two-tailed Student's *t*-tests were used to compare the transgenic lines and the corresponding controls.

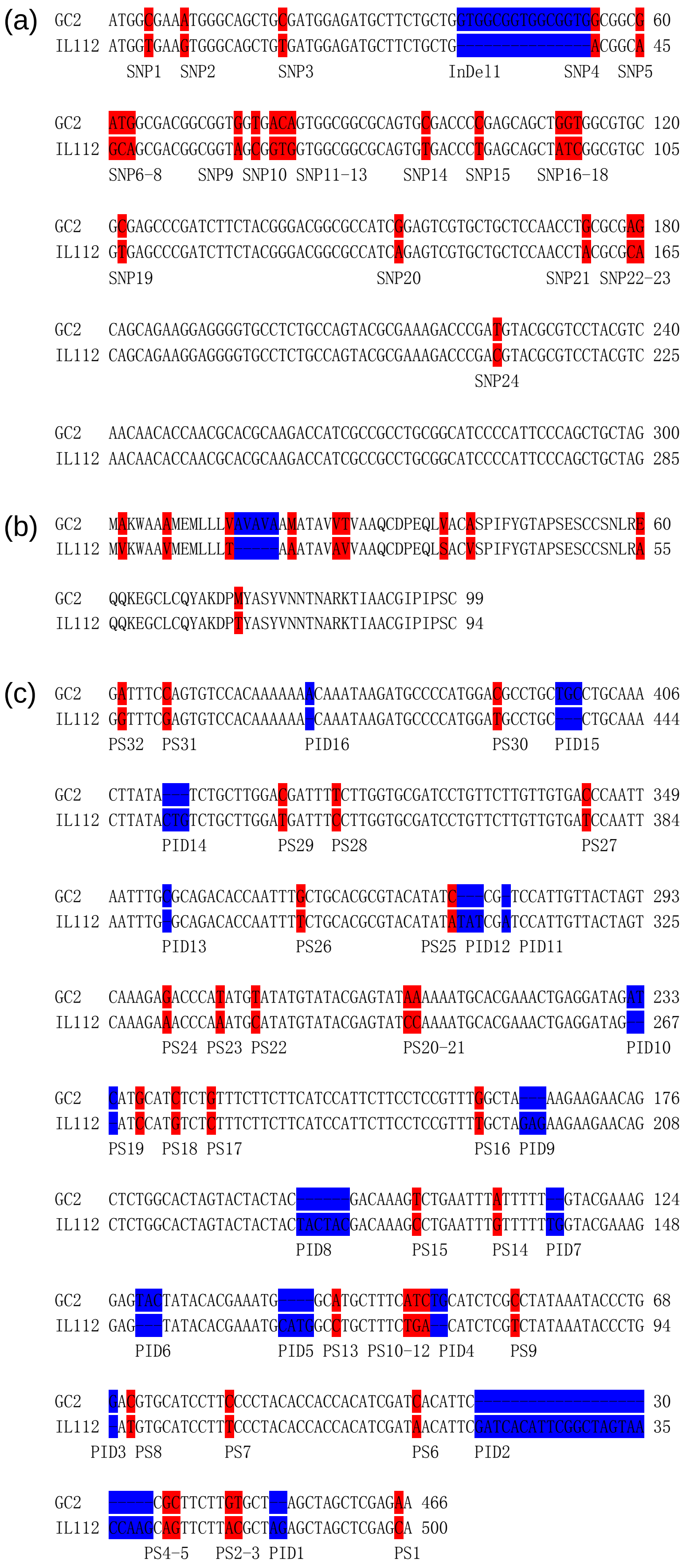

Figure S6. Sequence comparison of *OsLTPL159* between Guichao 2 (GC2) and IL112. (a) Comparison of the coding region sequence of *OsLTPL159* from GC2 and IL112. (b) Sequence alignment of the OsLTPL159 protein from GC2 and IL112. (c) Comparison of 500-bp 5′-flanking sequences of *OsLTPL159* from GC2 and IL112. The letters on the red background represent SNPs. The letters on the blue background represent insertions/deletions.

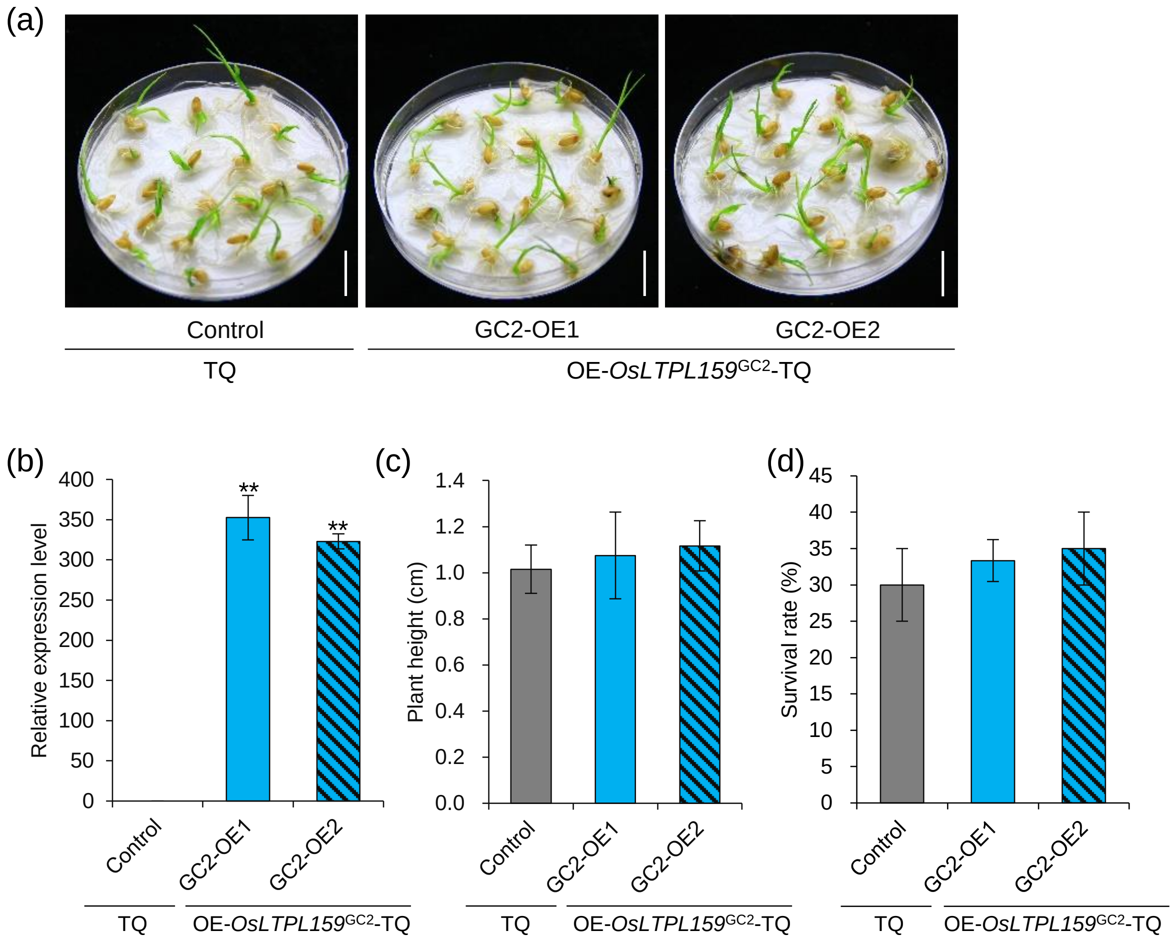

Figure S7. Evaluation of cold tolerance at the early seedling stage in transgenic lines overexpressing *OsLTPL159*^{GC2} from *indica* variety Guichao 2. (a) Phenotypes of the control line (TQ) and two overexpression transgenic lines (OE-*OsLTPL159*^{GC2}-TQ) after 6 d at 6 °C and a subsequent 7 d of recovery at 28 °C. Scale bars, 1.5 cm. (b) Comparison of *OsLTPL159* expression in the control (TQ) and OE-*OsLTPL159*^{GC2}-TQ transgenic lines. Values are means ± SD (n = 3 pooled tissues, 50 plants per pool). (c) and (d) Comparison of plant height (c) and survival rate (d) in the control (TQ) and OE-*OsLTPL159*^{GC2}-TQ transgenic lines after 6 d at 6 °C and a subsequent 7 d of recovery at 28 °C. Values are means ± SD (n = 3 replicates, 20 plants per replicate). Two-tailed Student's t-tests were conducted to compare the transgenic and control lines (**P < 0.01).
